# The future of the northeast Atlantic benthic flora in a high CO_2_ world

**DOI:** 10.1002/ece3.1105

**Published:** 2014-06-18

**Authors:** Juliet Brodie, Christopher J Williamson, Dan A Smale, Nicholas A Kamenos, Nova Mieszkowska, Rui Santos, Michael Cunliffe, Michael Steinke, Christopher Yesson, Kathryn M Anderson, Valentina Asnaghi, Colin Brownlee, Heidi L Burdett, Michael T Burrows, Sinead Collins, Penelope J C Donohue, Ben Harvey, Andrew Foggo, Fanny Noisette, Joana Nunes, Federica Ragazzola, John A Raven, Daniela N Schmidt, David Suggett, Mirta Teichberg, Jason M Hall-Spencer

**Affiliations:** 1 Department of Life Sciences, The Natural History MuseumCromwell Road, London, SW7 5BD, UK; 2 School of Earth and Ocean Sciences, Cardiff UniversityMain Building, Park Place, Cardiff, CF10 3YE, UK; 3 Marine Biological Association of the UKCitadel Hill, Plymouth, PL1 2PB, UK; 4 Ocean and Earth Science, National Oceanography Centre, University of SouthamptonWaterfront Campus, European Way, Southampton, SO14 3ZH, UK; 5 School of Geographical and Earth Sciences, University of GlasgowGlasgow, G12 8QQ, UK; 6 Marine Plant Ecology Research Group (ALGAE), Centre of Marine Sciences (CCMAR), University of AlgarveCampus of Gambelas, Faro, 8005-139, Portugal; 7 School of Biological Sciences, University of EssexColchester, CO4 3SQ, UK; 8 Institute of Zoology, Zoological Society of LondonRegent's Park, London, NW1 4RY, UK; 9 Department of Zoology, The University of British Columbia#4200-6270 University Blvd., Vancouver, British Columbia, V6T 1Z4, Canada; 10 DiSTAV - University of GenoaC.so Europa 26, Genoa, 16132, Italy; 11 Department of Earth and Environmental Sciences, University of St AndrewsSt Andrews, Fife, KY16 9AL, UK; 12 Scottish Oceans Institute, University of St AndrewsSt Andrews, Fife, KY16 8LB, UK; 13 Scottish Marine InstituteOban, Argyll, PA37 1QA, UK; 14 Institute of Evolutionary Biology, University of EdinburghThe King's Building, West Mains Road, Edinburgh, EH9 3JT, UK; 15 Institute of Biology, Environmental and Rural Sciences, Aberystwyth UniversityAberystwyth, UK; 16 Marine Biology and Ecology Research Centre, School of Marine Sciences and Engineering, Plymouth UniversityPL4 8AA, UK; 17 CNRS, UMR7144, Station Biologique de Roscoff, Place Georges Teissier, Roscoff Cedex, 29688, France; 18 UPMC Univ. Paris 6, UMR 7144Station Biologique de Roscoff, Place Georges Teissier, Roscoff Cedex, 29688, France; 19 Plymouth Marine LaboratoryProspect Place, The Hoe, Plymouth, PL1 3DH, UK; 20 School of Earth Sciences, University of BristolWills Memorial Building, Queen's Road, Bristol, BS8 1RJ, UK; 21 Division of Plant Science, University of Dundee at the James Hutton InstituteInvergowrie, Dundee, DD2 5DA, UK; 22 Plant Functional Biology and Climate Change Cluster, University of Technology SydneyUltimo, NSW 2007, Australia; 23 Leibniz-Zentrum für Marine TropenökologieFahrenheitstraße 6, Bremen, D-28359, Germany

**Keywords:** Calcified algae, climate change, invasive species, macroalgae, microphytobenthos, seagrasses, volatile gases

## Abstract

Seaweed and seagrass communities in the northeast Atlantic have been profoundly impacted by humans, and the rate of change is accelerating rapidly due to runaway CO_2_ emissions and mounting pressures on coastlines associated with human population growth and increased consumption of finite resources. Here, we predict how rapid warming and acidification are likely to affect benthic flora and coastal ecosystems of the northeast Atlantic in this century, based on global evidence from the literature as interpreted by the collective knowledge of the authorship. We predict that warming will kill off kelp forests in the south and that ocean acidification will remove maerl habitat in the north. Seagrasses will proliferate, and associated epiphytes switch from calcified algae to diatoms and filamentous species. Invasive species will thrive in niches liberated by loss of native species and spread via exponential development of artificial marine structures. Combined impacts of seawater warming, ocean acidification, and increased storminess may replace structurally diverse seaweed canopies, with associated calcified and noncalcified flora, with simple habitats dominated by noncalcified, turf-forming seaweeds.

## Introduction

Seaweed and seagrass communities in the northeast Atlantic have been profoundly impacted by humans, and the rate of change is accelerating rapidly due to runaway CO_2_ emissions, mounting pressures on coastlines associated with human population growth and increased consumption of finite resources. Global reviews of the known effects of global warming and ocean acidification (i.e., falling pH and carbonate levels combined with rising CO_2_ and bicarbonate levels) make it clear that although some taxa will benefit, others will be adversely impacted (Harley et al. 2012; Koch et al. 2013). Benthic phototrophs, that is, fleshy and calcified macroalgae, seagrasses, and microphytobenthos (MPBs), contribute significantly to coastal primary production, facilitate export of carbon from high to low productivity systems, and fuel entire food webs (Steneck et al. 2002). They also produce various volatiles, notably dimethyl sulfide (DMS) involved in algal physiology and defense (Stefels et al. 2007) that affect atmospheric chemistry and climate (Ayers and Cainey 2007; Carpenter et al. 2012). Species distributions are affected by a multitude of factors, but the major drivers of change are considered to be acidification and warming (Harley et al. 2012; Bijma et al. 2013). Some benthic algae and seagrasses are expected to thrive at higher CO_2_ levels, whilst others might be negatively impacted (Koch et al. 2013; Kroeker et al. 2013). High-latitude calcifying algae are at particular risk as surface waters are becoming more corrosive to their skeletons (Kamenos et al. 2013). Additionally, surface water warming is shifting the distributions of many species polewards (Poloczanska et al. 2013). The success of any photoautotroph in a high CO_2_ world will be a balance between its competitive ability for resources, resistance to herbivores, and tolerance to the environmental conditions (Connell et al. 2013).

Here, we make predictions as to how rapid warming and acidification (Feely et al. 2008; Steinacher et al. 2009) are likely to affect benthic flora and coastal ecosystems of the northeast Atlantic in this century based on global evidence from the literature as interpreted by the collective knowledge of the authorship. There has been considerable progress in our understanding of how primary producers are affected by changes in ocean temperature and acidification, but it is still unclear how this will affect ecosystems at the regional scale. Here, we focus on the northeast Atlantic as its long history of study provides a unique baseline from which to assess change (Brodie et al. 2009). The region supports a rich benthic flora including habitats formed by brown algae (e.g., kelp forests), coralline algae (e.g., carbonate deposits), and seagrass beds.

**Figure 1:**
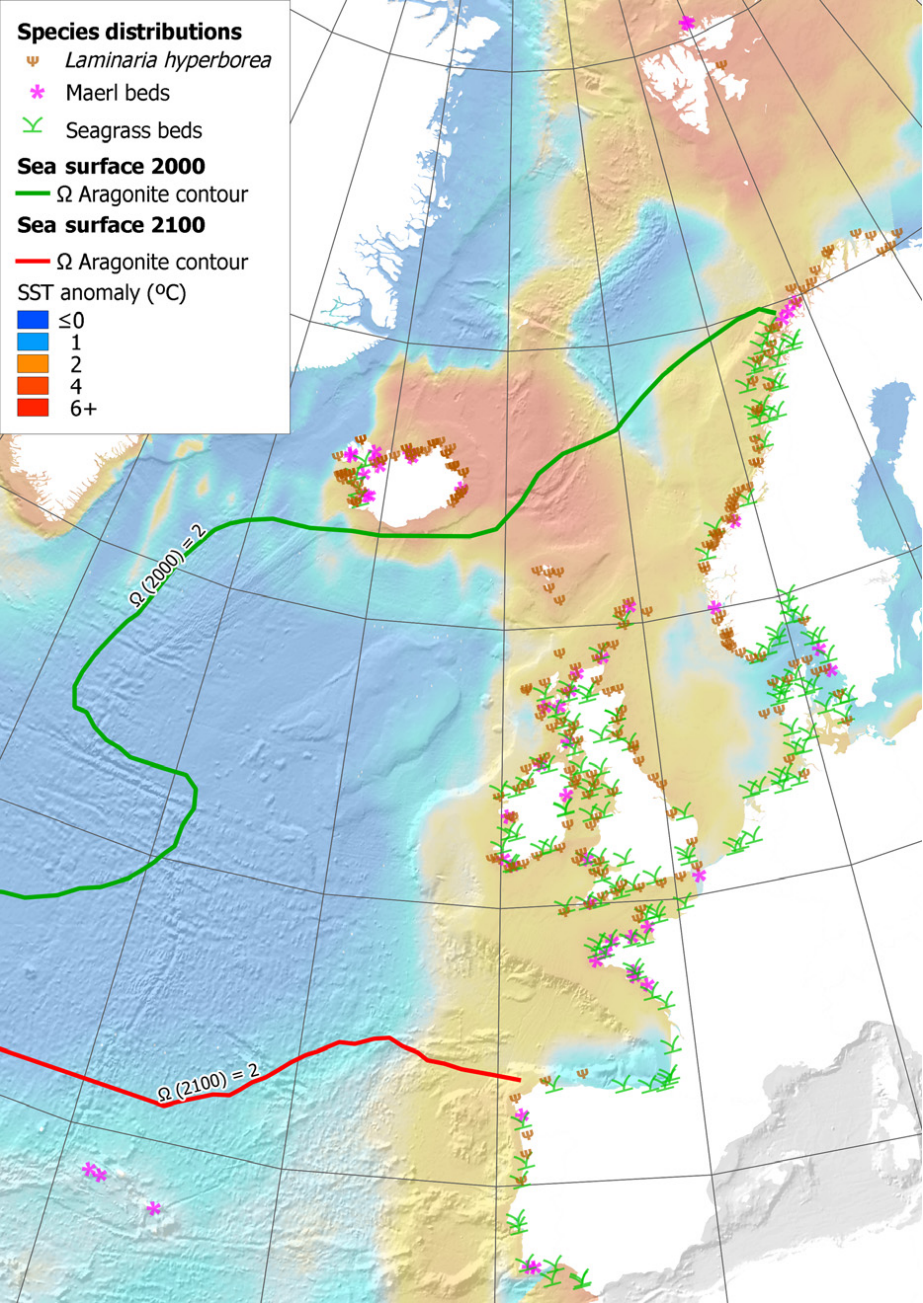
Present distribution of habitat-forming species in the northeast Atlantic, and an estimate of environmental change by 2100. SST anomaly (change from the present) is based on annual mean from an A1B scenario ensemble as Jueterbock et al. (2013). Many species' ranges such as the kelp *L. hyperborea* are thought to be limited by summer and winter thermoclines (van den Hoek 1982; Dieck 1993). Temperature changes are expected to impact distributions as species' ranges track these limits (Harley et al. 2012). Maerl are calcifying species utilizing high magnesium calcite, which has a similar saturation state to aragonite in the northeast Atlantic (Andersson et al. 2008). Most maerl are currently found in locations supersaturated for aragonite (Ω > 2). Predictions of the saturation state for 2100 (Steinacher et al. 2009) suggest that most of the northeast Atlantic will be outside this range.

Over the last century, human activities have had more impact on the coastal zone than climate change but whilst such human activities continue to increase (Nicholls et al. 2007 and refs therein) this is expected to change as sea surface isotherms are moving polewards rapidly in the northeast Atlantic whilst waters corrosive to carbonate are now present in shallow Arctic waters and are spreading south (Fig. 1).

**Figure 2:**
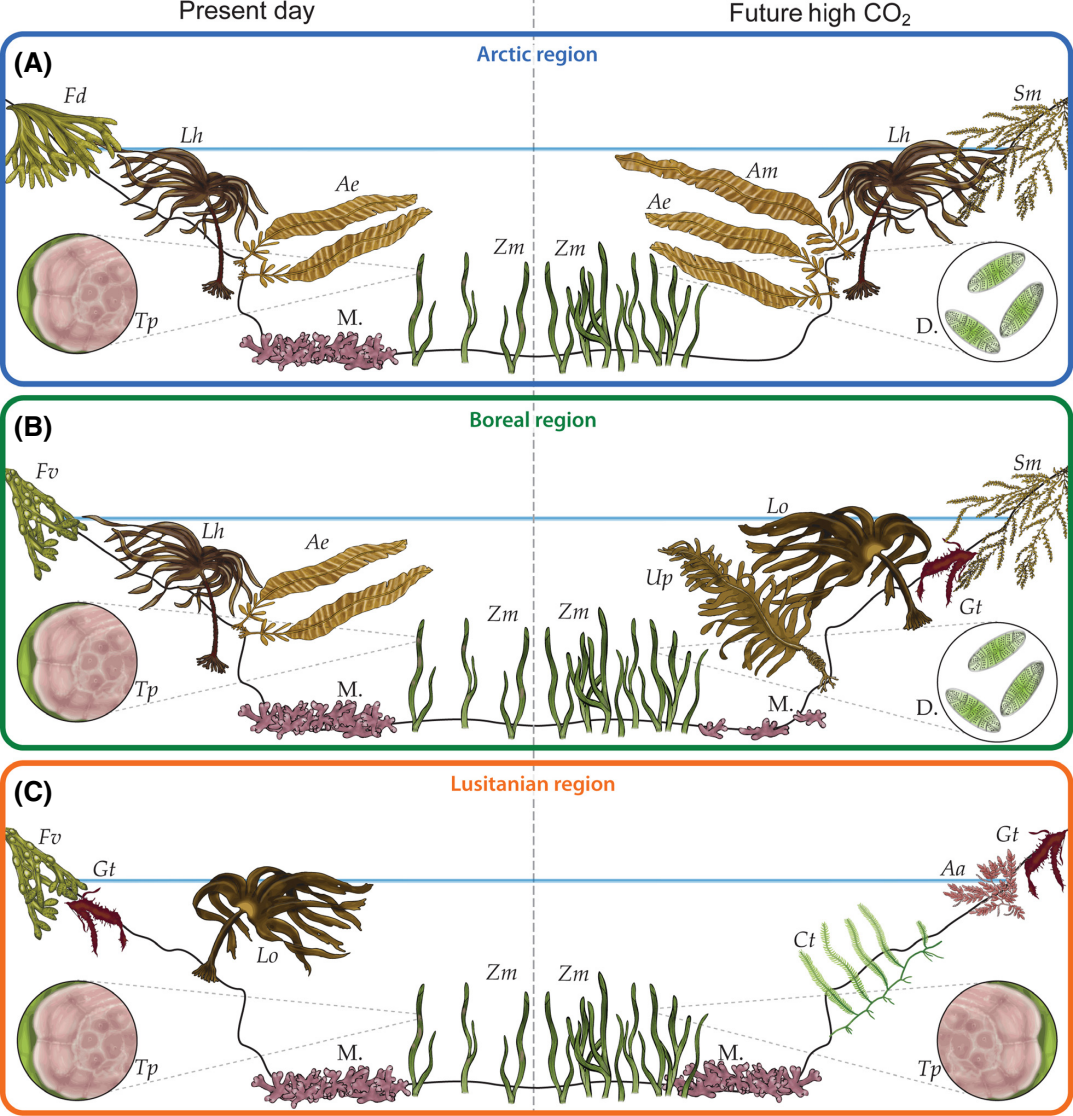
Predicted change in northeast Atlantic benthic marine flora if CO_2_ emissions continue unabated. (A) Arctic region: warming will be detrimental to cold-adapted species, and acidification will corrode maerl (M.). Pacific species, for example, *Alaria marginata* (Am), will invade as polar ice melts, competing with native species such as *Laminaria hyperborea* (Lh) and *Alaria esculenta* (Ae). Fleshy invasives, for example, *Sargassum muticum* (Sm), will move north competing with fucoids, for example, *Fucus distichus* (Fd), in the intertidal. Acidification will corrode epiphytic calcified algae, for example, *Titanoderma pustulatum* (Tp), and increased CO_2_ levels will stimulate growth of diatoms (D.) (magnified circles) and seagrasses such as *Zostera marina* (Zm). (B) Boreal region: *Laminaria hyperborea* (Lh) forests will be increasingly dominated by *Laminaria ochroleuca* (Lo), with the loss of *Alaria esculenta* (Ae) and fucoids, for example, *Fucus vesiculosus* (Fv) and the continued spread of invasive *Undaria pinnatifida* (Up), *Sargassum muticum* (Sm), and *Grateloupia turuturu* (Gt). As in the Arctic, maerl beds will be corroded, seagrasses will thrive, but epiphytic calcified algae will be reduced or replaced with diatoms and filamentous seaweeds (magnified circles). (C) Lusitanian region: kelps will be replaced by smaller, fleshy algae and invasive species, for example, *Caulerpa taxifolia* (Ct) will proliferate. Fucoids will be replaced by invasives such as *Asparagopsis armata* (Aa). Seagrasses will thrive, and it is expected that maerl and epiphytic calcified algae will be retained (magnified circles).

In this study, we review evidence and make predictions about the combined effect of warming and acidification on the following major groups of organisms: fleshy, invasive and calcified macroalgae, seagrasses, and MPBs. We capture the combined predictions in Figures 1 and 2 and, at the end, provide an outline of research that we consider needs to be undertaken. Our overall objective is to illustrate how these changes will affect the diverse and well-studied benthic marine flora of the northeast Atlantic and the impact on ecosystem structure and function. This should serve as a template to stimulate further discussion and work.

## Fleshy Algae

In the northeast Atlantic, kelp forests (Laminariales) dominate algal biomass in the subtidal and fucoids (Fucales) in the intertidal. Kelp beds are amongst the most productive habitats on Earth (Mann 1973, 2000; Reed et al. 2008) and are a major source of primary production in coastal zones of temperate and polar oceans worldwide (Steneck et al. 2002). Other fleshy algae, such as the large fucoids that dominate many intertidal habitats (e.g., *Ascophyllum nodosum)*, are also highly productive and play a key role in carbon capture and transfer in coastal ecosystems (Golléty et al. 2008). In the Atlantic, primary production can be 1000 g C m^−2^ year^−1^ for Laminariales and in excess of 500 g C m^−2^ year^−1^ for fucoids (Mann 1973, 2000; Vadas et al. 2004); this productivity represents a major component of coastal food webs. Whilst some macroalgal biomass is consumed directly by herbivorous fish and invertebrates, most biomass is processed as detritus or dissolved organic matter. Detrital biomass is then processed by microbes and may be consumed by suspension feeders, detrital grazers, and general consumers of organic material in soft sediments (deposit feeders), thereby transferring energy to higher trophic levels.

It is predicted, based on the relatively limited data available, that rising temperatures and ocean acidification will combine to profoundly alter fleshy algal species composition, abundance, and productivity worldwide (Harley et al. 2012; Krumhansl and Scheibling 2012; Koch et al. 2013). With continued warming, some species and populations will become chronically (gradual warming) or acutely (extreme events) stressed as temperatures exceed physiological thresholds. If physiological processes cannot be maintained, primary productivity will decrease and, ultimately, widespread mortality may ensue (Smale and Wernberg 2013), as evidenced by the retraction of kelp beds at their low latitudinal limits (Tuya et al. 2012; Wernberg et al. 2013). On the other hand, where waters remain cool enough, assemblages of fleshy macroalgae are expected to benefit from high CO_2_ conditions as increased inorganic carbon availability may enhance the growth and reproduction of fleshy macroalgae (reviewed in Harley et al. 2012; Koch et al. 2013; Kroeker et al. 2013). In Figure 2, we show examples of how such changes are predicted to affect the northeast Atlantic where the flora is dominated by kelps (Laminariales) in the subtidal and fucoids (Fucales) in the intertidal.

Such predictions are needed as kelp forests are amongst the most productive habitats on Earth and together with fucoids underpin the ecology of northeast Atlantic coastal ecosystems (Mann 1973; Smale et al. 2013). Algal communities are expected to increase in biomass, abundance, and detrital production in Boreal and Arctic waters in response to increased inorganic carbon availability as they lack calcified skeletons and so are immune to corrosion by acidified waters. We predict that North Pacific seaweeds, such as *Alaria marginata*, may colonize cooler regions of the northeast Atlantic (Fig. 2) due to warming and the opening of Arctic shipping routes. Species such as *Nereocystis luetkeana* are less likely to spread to the Atlantic as they are light limited at high latitudes and less easily spread via shipping. As kelps and fucoids are cool water adapted and stressed by high temperatures (Steneck et al. 2002), we predict that they will undergo significant changes in their distribution; there have already been widespread northeast Atlantic losses of the kelps *Saccharina latissima* (Moy and Christie 2012), *Saccorhiza polyschides*, *Laminaria ochroleuca* (Fernández 2011), *Laminaria hyperborea* (Tuya et al. 2012), *Laminaria digitata* (Yesson et al., unpublished manuscript), and *Alaria esculenta* (Simkanin et al. 2005; Mieszkowska et al. 2006; Merzouk and Johnson 2011) attributed to ocean warming in conjunction with other stressors. Of note, Bartsch et al. (2013) have highlighted that the main determinant in survival of *Laminaria digitata* from Helgoland was restricted temperature windows for sporogenesis due to sea surface temperature warming. Warming in the Boreal region is expected to replace *Laminaria hyperborea* with *L. ochroleuca*; this may have limited ecological impact, as these kelps are similar both structurally and functionally, although subtle differences in kelp structure can influence their associated communities (Blight and Thompson 2008).

There is considerable evidence of change in fucoid distribution in the northeast Atlantic. Range expansion in *F. vesiculosus* and no apparent change in distribution of *F. serratus* in Portugal (Lima et al. 2007) are countered by depleted genetic diversity in the latter species (Pearson et al. 2009; Jueterbock et al. 2013) and evidence of a significant decline for both species in the UK (Yesson et al., unpublished manuscript). Further evidence of decline in some regions includes *Ascophyllum nodosum* (Simkanin et al. 2005; Davies et al. 2007), *Pelvetia canaliculata* (Lima et al. 2007), *Chorda filum* (Eriksson et al. 2002), and *Himanthalia elongata* (Fernández and Niell 1982; Lima et al. 2007). We predict that there will be declines in the fucoids *Ascophyllum nodosum*, *Fucus serratus*, *F. vesiculosus* (Fig. 2), *Pelvetia canaliculata,* and the other large, common brown algae *Chorda filum* and *Himanthalia elongata* (Yesson et al., unpublished manuscript). We also predict that *Fucus distichus* will decline based on evidence of loss from its southern limit in the UK (Brodie et al. 2009).

In parallel, an increase in the relative abundance of fast-growing “annuals”, such as *Saccorhiza polyschides* and *Undaria pinnatifida*, is expected to have major implications for kelp forest structure and functioning, as stable perennial habitats become more “boom and bust” in nature (Smale et al. 2013). Whether or not a species is replaced by a functional equivalent could be key in future ecosystem functioning. For example, replacement of *Laminaria hyperborea* with *Laminaria ochroleuca*, which are similar both structurally and functionally, may have less impact, although *L. ochroleuca* does not support the diversity of stipe epiflora and fauna associated with *L. hyperborea*, and subtle differences in kelp species traits influence local biodiversity patterns (Blight and Thompson 2008).

In contrast, warming is expected to cause losses of the cool-temperate species *Alaria esculenta* in the Boreal region (Fredersdorf et al. 2009) which will alter ecosystems as it is the dominant species on very exposed shores and an important mid-successional species in more sheltered locations (Hawkins and Harkin 1985), yet there is no warm water equivalent to take its place.

As the northeast Atlantic continues to warm and acidify, we predict that kelp forests will die out in the Lusitanian region (Fig. 2). This shift from highly productive, large, structural kelp species to smaller fleshy or filamentous species is expected to decrease macrophyte biomass and detrital input to coastal food webs (Krumhansl and Scheibling 2012) with wide-ranging consequences for community structure and ecosystem functioning (Smale et al. 2013).

Both direct and indirect effects of changing water chemistry are likely to affect grazers and alter food webs (Alsterberg et al. 2013; Asnaghi et al. 2013; Borell et al. 2013; Falkenberg et al. 2013). Differences in algal defensive chemistry, structural properties, and nutritional quality in response to ocean acidification are likely to be manifest at both intra- and interspecific levels as resource allocation patterns (see Arnold and Targett 2003) and assemblages (see Kroeker et al. 2013) respond to reduced alkalinity; indeed, evidence already exists for the direct effects of acidification upon defenses and structure (e.g., Borell et al. 2013; Kamenos et al. 2013). Phaeophytes may be particularly implicated in cascading effects resulting from altered biochemistry in response to acidification as their carbon-dense phlorotannins, which can constitute 15% of algal dry mass (Targett et al. 1992), have reduced energetic production costs (see Arnold and Targett 2003) but are known to significantly influence both primary consumer and detritivore exploitation of algal tissues. Thus, both intrabenthic and benthic-pelagic trophic linkages are dependent upon the consumption of live and decaying seaweeds by primary consumers, processes mediated by acidity-sensitive algal characteristics (Hay et al. 1994).

## Invasive Species

The rate of recorded introductions of non-native algae and the spread of invasive algae are increasing in the northeast Atlantic (Arenas et al. 2006; Sorte et al. 2010), although direct evidence to indicate non-native benthic algae cause extinctions in communities is lacking (Reid et al. 2009). Approximately 44 species of non-native benthic macroalgae are reported for the northeast Atlantic (Guiry 2012) including large brown species such as *Sargassum muticum* and *Undaria pinnatifida*.

As with native species, those opportunistic invasive fleshy algae that are tolerant of warming and low carbonate saturation are likely to benefit from increased carbon availability (Weltzin et al. 2003). There is also evidence from a study of the invasive red seaweed *Neosiphonia harveyi* where the effects of low temperatures on photosynthesis were alleviated by increased *p*CO_2_ (Olischläger and Wiencke 2013) that suggests warmer water species will be able to move into cooler areas where calcareous algae and fleshy species such as the kelps and fucoids have been lost. At Mediterranean CO_2_ vents, invasive genera such as *Sargassum*, *Caulerpa,* and *Asparagopsis* thrive where native coralline algae are excluded by acidified waters (Hall-Spencer et al. 2008). Warming is expected to facilitate the spread of *Caulerpa taxifolia* into Lusitanian waters (Fig. 2), whilst northward range shifts of native fleshy species are expected to provide opportunities for invasive macroalgae to colonize. In Lusitanian regions, the die back of kelp forests due to increased temperatures may increase rates of macroalgal invasions by such species as *Asparagopsis armata* which is expected to proliferate alongside cooler water invasive species such as *Sargassum muticum*, *Undaria pinnatifida,* and *Grateloupia turuturu* in the Boreal region (Fig. 2).

Indirect changes associated with a high CO_2_ world will also likely impact the future dynamics of macroalgal invasions in the northeast Atlantic. As we switch to reliance on offshore renewable energy capture (Breton and Moe 2009), associated increases in new and artificial marine structures will likely provide important, competitor free, bare substrata, facilitating the spread, and establishment of non-natives (Nyberg and Wallentius 2005). Melting of the polar ice cap will also open up new invasion corridors between the Pacific and Atlantic Oceans in the form of both natural dispersion and introduction associated with polar shipping routes (Reid et al. 2007).

On the whole, we predict that under a high CO_2_ world, macroalgal invasions in the northeast Atlantic will increase, aided by increased carbon availability, increased stress imposed on native (especially calcareous) macroalgal species, loss of key habitat-forming kelps at their southerly limits, and indirect factors facilitating dispersal, transportation, and establishment of non-native populations.

## Calcified algae

There are a wide range of calcified taxa in the northeast Atlantic, including the red calcifying coralline algae, the green algal genus *Acetabularia,* and the brown algal genus *Padina*. The coralline algae include crustose coralline algae (CCA), free-living coralline algae (rhodolith/maerl), and geniculate (articulated) turfing algae. These form a cosmopolitan group of marine flora, ubiquitous in intertidal and shallow subtidal habitats, where they act as important ecosystem engineers (Kamenos et al. 2004; Nelson 2009).

As with fleshy algae, each calcified alga has a thermal optimum, so their distributions are probably already changing due to global warming and are expected to shift significantly as global sea surface temperatures continue to rise. Furthermore, calcified algae may not benefit from the increasing availability of inorganic carbon for photosynthesis as ocean acidification also increases the metabolic costs of calcification and can corrode their skeletons when carbonate becomes undersaturated (Nelson 2009).

We predict that one of the largest impacts of sustained CO_2_ emissions will likely be the dissolution of areas of dead maerl and to a lesser extent live maerl habitat in the northeast Atlantic. Surface water that is corrosive to algal carbonate is already expanding southwards in the Arctic (Steinacher et al. 2009). Although there is conflicting laboratory evidence over the vulnerability of live maerl to future conditions (Noisette et al. 2013), field observations show that maerl beds mainly form in waters with high carbonate saturation (Hall-Spencer et al. 2010). Although some coralline algae sustain calcification over long periods of exposure to elevated *p*CO_2_, a loss of structural integrity is inherent (Ragazzola et al. 2012; Kamenos et al. 2013; Martin et al. 2013), which presumably comes with an energetic cost to growth (Bradassi et al. 2013). Those species that require stable conditions at high carbonate saturation states are likely to be negatively impacted (Büdenbender et al. 2011). We expect that maerl habitat will be lost at high latitudes as aragonite saturation falls (Fig. 1), although Lusitanian maerl will persist (Fig. 2). As thin epiphytic coralline algae dissolve easily (Martin et al. 2008), they are expected to decline in areas where seawater becomes corrosive to their skeletons. Those species that tolerate widely fluctuating levels of CO_2_, such as intertidal *Corallina* and *Ellisolandia* species, will be more resilient to ocean acidification (Egilsdottir et al. 2013). However, competition from fleshy algal species that benefit from high CO_2_ may indirectly lead to loss of calcified species (Kroeker et al. 2013). Similarly, persistence of species in decalcified forms under high CO_2_ may contribute to phase shifts from calcified dominated assemblages to fleshy algae (Johnson et al. 2012).

Northeast Atlantic coralline algal habitats are reported to contain more than double the annual open-ocean average of dissolved DMS concentration (Burdett 2013); thus, loss of calcified algae, in combination with biogeographic shifts and species invasions, may alter habitat taxonomic composition to low-DMSP-producing fleshy algae (Fig. 2). The loss of structural integrity of coralline algal skeletons under high CO_2_ conditions may also facilitate the release of DMSP into the surrounding water column, stimulating the microbial consumption of DMSP and production of DMS (Burdett et al. 2012).

Overall, we predict there may be significant loss of primarily dead but also living calcified macroalgae in the northeast Atlantic by 2100, beginning at high latitudes and spreading further south over the century. Monitoring is required to assess the impact of these changes given the importance of calcified algae to fisheries and ecosystem function (Kamenos et al. 2013).

## Seagrasses

Extensive seagrass beds are found in the northeast Atlantic (Fig. 1). They sequester carbon through photosynthesis and store large quantities in both the plants, but more importantly, in the sediment below them (Mcleod et al. 2011; Fourqurean et al. 2012). Unlike rainforests where the carbon captured remains for decades or centuries, the carbon captured by sediments from seagrasses can remain stored for millennia (Mateo et al. 1997).

At present, seagrasses are carbon limited and are thus expected to benefit from ocean acidification due to increased available substrate for photosynthesis. Therefore, considering the carbon sequestration ability of seagrasses and predicted increases in inorganic carbon utilization due to ocean acidification (Koch et al. 2013), we predict that in a high CO_2_ world the below-ground carbon pool associated with northeast Atlantic seagrass beds will increase. Paleoreconstruction of sediments underlying old seagrass meadows may reveal the long-term carbon sequestration patterns of northeast Atlantic seagrass species (Mateo et al. 2010) and allow future predictions.

Although loss of seagrass' calcareous epiphytes may be beneficial through removal of associated oxidative stress, under high CO_2_, nutrients and temperature, we predict that non-calcareous epiphytes such as filamentous algae and diatoms will increase (Alsterberg et al. 2013). This may lead to shifts in the epiphyte community structure from less palatable calcareous, to more palatable algae. Additionally, decreased production of grazing deterrent phenolics by seagrasses under high CO_2_ (Arnold et al. 2012) may increase the palatability of seagrass leaves for a number of invertebrate and fish grazers, maintaining or increasing grazing rates of seagrass blades, depending on food preferences of grazers and the availability of other food sources.

Positive effects of increased CO_2_ on seagrass physiology may help to ameliorate negative effects of other environmental stressors known to impact seagrass growth and survival. If seagrasses are afforded the protection they need from damage by fishing gear, dredging, and both organic and nutrient pollution, we predict these habitats will proliferate in a high CO_2_ northeast Atlantic, albeit with the loss of certain calcified organisms and the increasing spread of invasive macroalgae within seagrass habitats (Fig. 2).

## Microphytobenthos

The microphytobenthos (MPBs) are benthic microscopic algae including cyanobacteria, diatoms, benthic dinoflagellates, and diminutive life-history stages of macroalgae. They are the base of many food webs, sustaining thousands of species of grazing and deposit feeding invertebrates in the northeast Atlantic, and they form biofilms that affect the colonization of rocky substrata, the biogeochemistry of sediments, and stabilize coastal mud flats. Some MPBs effectively exist via symbiotic relationships with invertebrates such as anemones and corals whilst other MPBs live within shellfish and can be severely toxic to humans.

We predict that there will be an increasing abundance of diatoms in northeast Atlantic MPB, based on evidence from studies conducted at CO_2_ vent sites in the Mediterranean Sea where most insight into the potential impacts of high CO_2_ on the MPB come from. In these vent systems, diatom- and cyanobacteria-dominated biofilms predominate, and broad scale analysis of microeukaryote diversity has shown that MPB communities in high CO_2_ water are substantially modified compared with ambient conditions (Lidbury et al. 2012). Responses to elevated CO_2_ are, however, variable between different diatom and cyanobacteria groups (Raven et al. 2012; Johnson et al. 2013). The response of toxic dinoflagellates to high CO_2_ conditions should also be considered in the northeast Atlantic, given previous switches to toxic bloom states observed in paleo/fossil records (Sluijs et al. 2007), evidence of shift toward less toxic variants under high CO_2_ (Eberlein et al. 2012), and the potential for enhanced production of toxins during high CO_2_ conditions (Fu et al. 2010).

Due to potential increased carbon uptake by MPB, it is also possible to predict an increased export of organic carbon and subsequent production of an extracellular biofilm matrix, as has been observed under high CO_2_ conditions at the Volcano vents (Lidbury et al. 2012), and in analogous planktonic systems (Borchard and Engel 2012). Given that MPBs, with seagrasses, determine sediment organic matter composition (Hardison et al. 2013), increased carbon export by CO_2_-stimulated MPB could significantly alter carbon cycling processes across northeast Atlantic sediment ecosystems. However, OA also increases degradation of polysaccharides by bacterial extracellular enzymes (Piontek et al. 2010), indicating that OA-controlled feedback mechanisms will occur.

To allow further predictions, we require a deeper understanding of the mechanistic effects of high CO_2_ on key MPB groups. This will require research into dissolved inorganic carbon (DIC) uptake-mechanisms and intracellular pH regulatory mechanisms. The production of CO_2_ internally from active uptake of 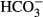 or externally via carbonic anhydrase activity will be strongly influenced by intracellular and cell surface pH (Taylor et al. 2011; Flynn et al. 2012). Additionally, cell size, shape, and biofilm formation can have profound effects on cell surface pH relations and consequent DIC speciation. pH at the surface of larger cells or aggregates is influenced significantly more by metabolic membrane H^+^ fluxes, with substantial cell surface pH fluctuation in relation to photosynthetic metabolism observed for large diatom cells (Kühn and Raven 2008; Flynn et al. 2012). Under elevated CO_2_, larger cells are likely to experience substantially larger diurnal pH fluctuations than smaller cells (Flynn et al. 2012). A deeper understanding of the direct effects on physiology will be critical in order to model impacts of elevated CO_2_ on MPB.

In addition, MPB responses to high CO_2_ need to be understood at the ecosystem level. For example, biogeochemical impacts of CO_2_ enhanced MPB communities may be modulated by heterotrophic components of the same community (Witt et al. 2011), or increased MPB biomass may be mediated by grazing pressure (Alsterberg et al. 2013). In the northeast Atlantic, the impacts of OA on MPB community diversity could further modify, or be modified by, other impacts such as increased temperature and eutrophication.

## Conclusions

Carbon dioxide emissions are causing rates of global warming and ocean acidification that will profoundly affect marine flora worldwide (Pörtner et al. 2014). We have illustrated how these changes will affect the diverse and well-studied benthic marine flora of the northeast Atlantic (Figs. 1 and 2), and how these changes will likely affect ecosystem structure and function. It is clear that unless CO_2_ emissions are curbed, there will be far-reaching consequences for regional biodiversity patterns, trophic linkages, nutrient cycling, and habitat provision for socio-economically important marine organisms. Warming will kill off kelp forests in the south, and ocean acidification will remove maerl habitat in the north. Seagrasses will proliferate, and associated epiphytes switch from calcified algae to diatoms and filamentous species. Invasive species will thrive in niches liberated by loss of native species and spread via exponential development of artificial marine structures. Thus, combined impacts of seawater warming, ocean acidification, and increased storminess may replace structurally diverse seaweed canopies with associated calcified and noncalcified flora with simple habitats dominated by noncalcified, turf-forming seaweeds.

Over the longer term, the ability and rate of species/populations to evolve will be crucial (Sunday et al. 2014). Evolutionary change may lead to adaptation, but it still may not be enough to prevent extinctions due to warming and acidification (Lohbeck et al. 2012). It will be vital to understand and measure predictors of evolution, such as genetic variability within and between populations, and to understand how knowledge of plastic responses can be leveraged to predict the evolutionary and/or adaptive potential of populations. A much greater effort is needed to develop real time maps of the key populations and their genetic diversity.

Future research must also address the impact that loss of the calcified and fleshy algae and their habitats will have on other benthic flora groups, and benthic, pelagic, and terrestrial fauna that are dependent on such resources. The responses of MPB assemblages, and species-specific information for DMSP and DMS production in algae and seagrasses that will form the benthic floral assemblages under increased CO_2,_ are required. Underpinning this is a need to quantify natural variability in carbonate chemistry in the northeast Atlantic to gain a complete understanding of the carbonate chemistry environment experienced by species.

Finally, unless we take action, we will sleepwalk through radical ecological changes to the phycology of our coasts.
